# Development and Validation of a New Type of Displacement-Based Miniatured Laser Vibrometers

**DOI:** 10.3390/s24165230

**Published:** 2024-08-13

**Authors:** Ke Yuan, Zhonghua Zhu, Wei Chen, Weidong Zhu

**Affiliations:** 1 Department of Mechanical Engineering, University of Maryland, Baltimore County, Baltimore, MD 21250, USA; kyuan1@umbc.edu; 2 OmniSensing Photonics LLC, Columbia, MD 21046, USA; zhonghua.zhu@osphotonics.com (Z.Z.); wei.chen@osphotonics.com (W.C.)

**Keywords:** miniatured laser vibrometer, integrated optics, quadrature demodulation network, vibration and ultrasonic vibration measurements

## Abstract

Developing a miniatured laser vibrometer becomes important for many engineering areas, such as experimental and operational modal analyses, model validation, and structural health monitoring. Due to its compact size and light weight, a miniatured laser vibrometer can be attached to various mobilized platforms, such as an unmanned aerial vehicle and a robotic arm whose payloads can usually not be large, to achieve a flexible vibration measurement capability. However, integrating optics into a miniaturized laser vibrometer presents several challenges. These include signal interference from ghost reflectance signals generated by the sub-components of integrated photonics, polarization effects caused by waveguide structures, wavelength drifting due to the semiconductor laser, and the poorer noise characteristics of an integrated laser chip compared to a non-integrated circuit. This work proposes a novel chip-based high-precision laser vibrometer by incorporating two or more sets of quadrature demodulation networks into its design. An additional set of quadrature demodulation networks with a distinct reference arm delay line length can be used to conduct real-time compensation to mitigate linear interference caused by temperature and environmental variations. A series of vibration measurements with frequencies ranging from 0.1 Hz to 1 MHz were conducted using the proposed laser vibrometer to show its repeatability and accuracy in vibration and ultrasonic vibration measurements, and its robustness to test surface conditions. The proposed laser vibrometer has the advantage of directly measuring the displacement response of a vibrating structure rather than integrating its velocity response to yield the measured displacement with a conventional laser Doppler vibrometer.

## 1. Introduction

A laser Doppler vibrometer (LDV) can measure the velocity response of a vibrating structure based on the Doppler shift principle. As a noncontact vibration measurement device, it can avoid mass loading and is more suitable than a contact-type device, such as an accelerometer and a linear variable differential transducer, for measuring the vibration of a light-weight and fragile structure. Scanning LDV and continuously scanning LDV [1,2] techniques were developed to make LDV measurements automated, rapid, and dense. Experimental and operational modal analyses using an LDV have been widely used in engineering areas, such as model validation, modal identification, structural health monitoring, and damage detection of beams [3], plates [4], pipes [2,5], bridges [6,7], buildings [8,9], turbine blades [1,10], and sandwich structures [11,12].

With the development of unmanned system techniques, vibration measurements by attaching an LDV on an unmanned system, such as an unmanned aerial vehicle (UAV) [13,14] and a robotic arm [15,16,17,18], are becoming more and more popular. By taking the LDV off a tripod, which provides a common support for the LDV, vibration measurement can be more flexible and practical. However, for such UAV+LDV and Robot+LDV systems, one of the major challenges was the limitations of the payloads of the UAV and the robot, as higher payloads indicated higher costs and risks during operations. In Ref. [14], the UAV with a sufficiently large payload was selected to hold both the sensor head and the LDV module. In most of the above Robot+LDV systems, industrial robotic arms were used to hold one or multiple laser heads, which needed large spaces for operations and were not practical for most laboratory scenarios. Possible solutions for reducing robot payloads included separating a laser head and its accessories such as a laser excitation device [17], and using an LDV with a sufficiently small size.

This work aims at developing a new type of miniatured displacement-based laser vibrometers based on integrated optics to resolve the payload limitation issue. Most recent LDVs are designed using discrete optical components. The difference between discrete and integrated optical designs in LDVs can be compared to the difference between vacuum tube circuit and integrated circuit technologies in the electronics world. Main compact LDV techniques include photonic integrated circuits (PICs), laser feedback interferometry, and micro-machined free-space optical interferometers [19,20,21]. Ref. [20] presented a design on a silicon-on-insulator (SOI) platform for measuring small vibrations at low frequencies. Integrated optics can put an entire optical system into a single chip to reduce its size and weight by thousands of times. Apart from significantly reducing power consumption, size, weight, and cost, integrated optics can offer the following benefits. First, integrated optics employs semiconductor manufacturing processes to consolidate various optical elements and functions onto the chip, minimizing manual assembly steps. This approach can effectively reduce the human error, improve the overall manufacturing yield of LDVs, and enhance its long-term stability and reliability. Second, semiconductor manufacturing techniques offer exceptional accuracy, enabling the fabrication of optical devices at the nanometer scale. This level of precision supports the creation of optical systems with superior accuracy. Optical waveguides, filters, and other components can be produced and seamlessly integrated into the chip with unparalleled precision and consistency, compared to conventional discrete optical systems. Third, a modular design permits the standardization and reuse of various functional units, such as waveguides, beamsplitters, filters, and modulators or demodulators, across multiple functional blocks. This flexibility promotes scalability and functional expansion. The reuse of standardized components accelerates design iteration and streamlines the entire optical system design process. However, integrating optics into a miniaturized laser vibrometer presents several challenges. These include signal interference from ghost reflectance signals generated by the sub-components of integrated photonics, polarization effects caused by waveguide structures, wavelength drifting due to the semiconductor laser, and the poorer noise characteristics of the integrated laser chip compared to a non-integrated circuit.

To address the above limitations and challenges, this work proposes a novel chip-based high-precision laser vibrometer by incorporating two or more additional sets of quadrature demodulation networks into its design. Major contributions of this work include: (1) an additional set of quadrature demodulation networks with a distinct reference arm delay line length that can mitigate nonlinear interference caused by temperature and environmental variations by conducting real-time compensation; (2) the proposed laser vibrometer can directly measure the vibration displacement of a structure using a phase demodulation method, which is different from a frequency demodulation method used for a conventional LDV, which directly measures the vibration velocity and integrates it to obtain the displacement; and (3) the proposed laser vibrometer has good robustness to test surface conditions.

The remainder of this paper is organized as follows. Section 2 describes the working principles of laser interferometers, a quadrature coherent detector, and the laser vibrometer proposed in this work. Section 3 shows the performance of the proposed laser vibrometer based on its working principles to demonstrate its feasibility in production. Section 4 demonstrates a series of vibration measurements with frequencies ranging from 0.1 Hz to 1 MHz using the proposed laser vibrometer to show its repeatability and accuracy in vibration and ultrasonic vibration measurements, and its robustness to test surface conditions. Section 5 concludes the paper.

## 2. Working Principles Used in This Work

### 2.1. Basic Working Principles of Laser Interferometers

**Figure 1:**
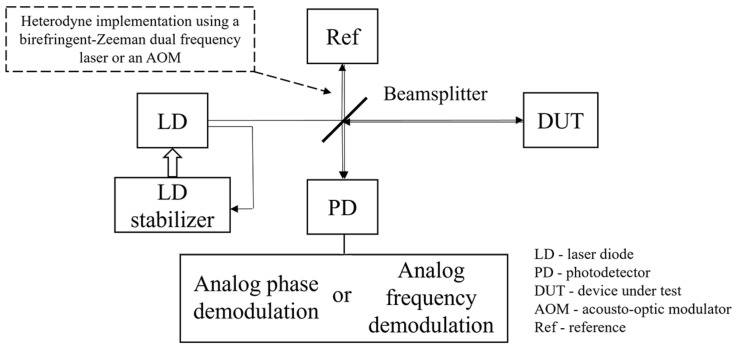
Simplified schematic diagram of a laser interferometer: the homodyne configuration without heterodyne implementation marked by the dashed box, and the heterodyne configuration.

A laser Doppler vibrometer is developed based on the working principles of laser interferometry. A simplified schematic diagram of a typical laser interferometer is shown in Figure 1. A laser diode (LD) with a narrow linewidth is usually preferred as the light source to ensure laser coherence and low phase noise, and an LD stabilizer unit can be used to stabilize its frequency. A beamsplitter is used to divide the laser beam into two: one as the measurement beam and the other as the reference beam. The measurement beam, after reflecting from a device under test (DUT), recombines with the reference beam to generate a beating signal that can be received by a photodetector (PD). Based on the relation between the frequency of the measurement beam and that of the reference beam, laser interferometers can be categorized as homodyne laser interferometers and heterodyne laser interferometers. In a homodyne laser interferometer, the two laser beams have the same frequency, and the displacement of the DUT can be obtained by analyzing the frequency or the phase shift of the beating signal. In a heterodyne laser interferometer, the two laser beams have different frequencies, which can be implemented by using a birefringence-Zeeman dual frequency laser as the laser source or by introducing an acousto-optic modulator (AOM) into its reference arm, as shown in the dashed box in Figure 1. The response of the DUT can then be obtained by analyzing the frequency or the phase shift of the beating signal. The beating signal with a high frequency, which is typically in the range of MHz, can effectively isolate the measured response of the DUT from environmental noise to preserve the measurement precision, even under suboptimal conditions. Heterodyne interferometers move signal frequencies to the MHz range while homodyne interferometers have signal frequencies to remain at original frequencies, which results in the fact that heterodyne interferometers are less susceptible to low-frequency noises than homodyne interferometers. There are several low-frequency noises. One is the 1/f noise, where *f* is the driver frequency, from the electronics that typically affect the LD driver and causes a high signal noise level in the low-frequency range. Also, optical components are susceptible to temperature change, which is usually slow and becomes another major noise source in the low-frequency range. Consequently, heterodyne interferometers have a better noise performance. Therefore, strong resistance to environmental noise is one of the major advantages of a heterodyne laser interferometer over a homodyne laser interferometer whose reference optical path is susceptible to environmental disruption in a non-integrated setting.

There are two methods to demodulate the vibration signal received by the PD. The first one is the phase demodulation method. It employs the demodulation of the amplitude changes of the received signal or filtering to directly obtain the envelope of the signal, thereby extracting the changes of the signal phase. The second one is the frequency demodulation method. Devices such as lock-in amplifiers can accurately detect changes in the frequency of the signal. Frequency shifts indicate the changes in the physical quantity being measured. However, in this configuration, the PD loses the complex signal information carried by photons in converting the received light power into the electrical signal. The most straightforward benefit of a complex baseband architecture is the noise-figure improvement achievable by eliminating the imaginary band noise foldback. In theory, the noise-figure improvement is 3 dB [22]. Environmental changes, mainly the thermal effect, result in the design parameter variations of various optical components, especially for an integrated photonic chip. With a complex domain signal, these changes can be compensated in digital decoding [23]. Therefore, the amplitude signal is highly susceptible to system noise and environmental noise. The frequency demodulation method is more precise under conditions with low signal-to-noise ratios (SNRs), and most commercial laser vibrometers are essentially heterodyne laser interferometers using the frequency demodulation method, earning them the name LDVs.

### 2.2. Working Principle of a Quadrature Coherent Detector

**Figure 2:**
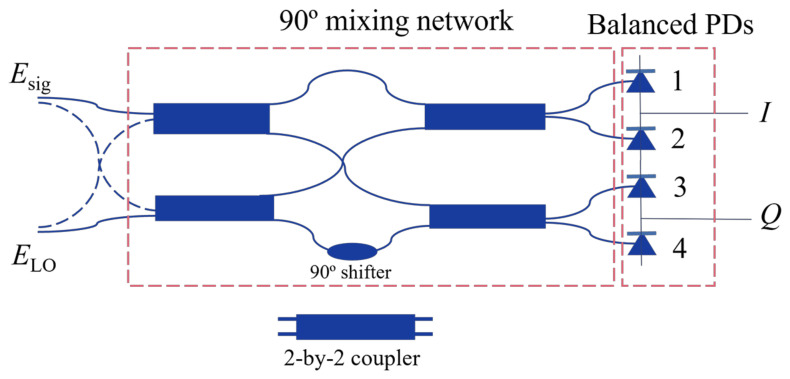
Schematic of quadrature demodulation.

Although the homodyne laser interferometer using the phase demodulation method is susceptible to noise, it is more suitable than the heterodyne laser interferometer for manufacturing a photonic integrated miniatured laser vibrometer due to its simpler configuration. In order to greatly enhance the performance of the homodyne laser interferometer in conditions with low SNRs, quadrature demodulation can be adopted in the design to recover the complete complex-domain photon signal. A schematic of quadrature demodulation is shown in Figure 2. An incoming signal beam can be split into an in-phase component (*I*) and a quadrature component (*Q*) through the beamsplitter in a 2-by-2 coupler. Similarly, a reference signal generated by a local oscillator (LO) laser can be split into two beams: one beam maintains its original phase for mixing with the in-phase component, while the other undergoes a 90° phase shift to mix with the quadrature component. Therefore, quadrature demodulation can also be referred to as a 90° mixing network. Four outputs from the 90° mixing network can be expressed as

(1) $$E_{1}=\frac{1}{2}\left(E_{\mathrm{sig}}+E_{\mathrm{LO}}\right),$$

(2) $$E_{2}=\frac{1}{2}\left(E_{\mathrm{sig}}-E_{\mathrm{LO}}\right),$$

(3) $$E_{3}=\frac{1}{2}\left(E_{\mathrm{sig}}+iE_{\mathrm{LO}}\right),$$

(4) $$E_{4}=\frac{1}{2}\left(E_{\mathrm{sig}}-iE_{\mathrm{LO}}\right),$$

where $i=\sqrt{-1}$, and $E_{\mathrm{sig}}$ and $E_{\mathrm{LO}}$ are the signals of measurement and reference beams, respectively. Mixed signals can then be received by balanced PDs that can eliminate common-mode noise, yielding enhanced SNRs. Finally, a signal processing unit receives *I* and *Q* signals, and demodulates their amplitude and phase information, allowing for the precise detection of the input signal, which can be written as

(5) $$I=R\sqrt{P_{\mathrm{sig}}P_{\mathrm{LO}}}\cos\left(\theta_{\mathrm{sig}}\left(t\right)-\theta_{\mathrm{LO}}\left(t\right)\right),$$

(6) $$Q=R\sqrt{P_{\mathrm{sig}}P_{\mathrm{LO}}}\sin\left(\theta_{\mathrm{sig}}\left(t\right)-\theta_{\mathrm{LO}}\left(t\right)\right),$$

where *R* denotes the responsivity of PDs, $P_{\mathrm{sig}}$ and $P_{\mathrm{LO}}$ denote the amplitudes of the signals of measurement and reference beams, respectively, and $\theta_{\mathrm{sig}}$ and $\theta_{\mathrm{LO}}$ denote the phases of of the signals of measurement and reference beams, respectively.

Incorporating quadrature demodulation can significantly enhance the performance of demodulation by the following aspects. The first one is to enhance phase accuracy. The 90° mixing network introduced above can produce two signals (*I* and *Q*) with a 90° phase difference, thereby delivering comprehensive phase information. Compared to conventional amplitude-phase demodulation methods, such as a fringe counting method, the *I*/*Q* phase demodulation method uses digital decoding, which can significantly improve the precision of measurement through subdivision. It ensures that the complete phase information can be accurately reconstructed by a complementary path when a signal path is disrupted by noise or partial distortion. The second one serves to resolve phase ambiguity. In a conventional single-frequency interferometer, phase measurement is typically limited to the range of [0,2π], which can lead to phase “folding” or “wrapping” beyond this range and cause ambiguity. The 90° mixing network can resolve this issue by accurately computing the angle of two orthogonal signals, enabling accurate and continuous phase determination even beyond the range of [0,2π]. Thus, the two orthogonal signals offer a more complete view of phase changes, expanding the dynamic range of measurement. This allows the interferometer to accurately measure the displacement or optical path difference variations over a wider range by digital decoding from a block of data. The last one is to reduce impacts from signal errors and noises. With two independent signal channels processed concurrently, specialized algorithms, such as trending detection and signal averaging, can minimize random noises and enhance the laser vibrometer’s responsiveness to genuine signals. This data processing helps cancel the nonlinear errors induced by a variety of noise sources or environmental fluctuations.

### 2.3. Working Principle of the Proposed Laser Vibrometer

**Figure 3:**
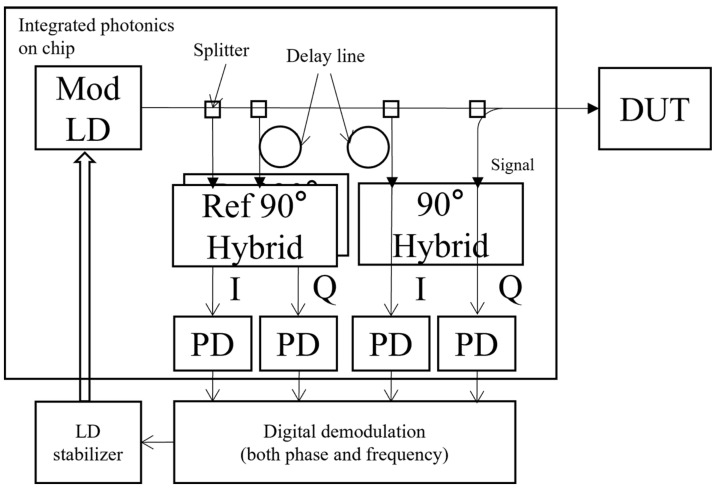
Schematic of the proposed chip-based high-precision laser vibrometer.

It is usually difficult to implement quadrature demodulation in a discrete optical device because it requires an accurate 90° delay on one interference beam in the demodulation network. However, it is relatively easy for photonic integration as the modern lithography processing can accurately control the length of the waveguide. A schematic of the proposed chip-based high-precision laser vibrometer is shown in Figure 3. The laser vibrometer proposed in this work incorporates two or more sets of quadrature demodulation networks in the design. One of the quadrature demodulation networks serves as a main *I*/*Q* demodulator for detecting the interference signal between measurement and reference beams and enabling the extraction of the phase and amplitude information from the signal beam. A second quadrature demodulation network serves as a reference *I*/*Q* demodulator for capturing the phase and amplitude instability of the laser. The demodulated reference signal is used for a closed control loop to lock the laser wavelength, and thus ensure signal stability in the laser vibrometer. The reflection signal from the DUT is only fed into the first quadrature demodulation network, and is mixed with the local LD source; *I*/*Q* signals are then decoded. The input signal of the second quadrature demodulation network is a split LD signal from the LD source after a delay line within the integrated photonic chip. This second quadrature demodulation network is used to catch the interference signal by slow laser center frequency drifting due to temperature change. The signal is considered as the common mode error and deducted by decoded results from the first quadrature demodulation network to retrieve an accurate signal from the DUT. As the delay line of the second quadrature demodulation network is co-printed with the delay line of the first quadrature demodulation network and the path lengths of the second quadrature demodulation network match with those of the first quadrature demodulation network, the common mode error can be removed by the proposed design. The novel laser vibrometer proposed in this work employs a fully digital demodulation approach to capture the comprehensive complex signal information. The reference *I*/*Q* demodulator stabilizes the frequency of the semiconductor laser through real-time feedback, reducing phase noise. This strategy allows the regular semiconductor laser with a prone-drifting center wavelength to achieve the performance level comparable to the conventional helium-neon gas laser. In addition, the modern semiconductor laser has a lower laser linewidth and can be easily integrated into a photonic chip.

However, there are also many challenges to integrate all the optics on the chip. First, insertion loss can be imbalanced in the component design, which can lead to unequal signal losses across different interference arms. Thus, the *I*/*Q* demodulation network is not ideal, which needs to be compensated in digital processing. In addition, internal reflection and scattering within waveguide components can cause unwanted optical signal interference, greatly complicating the demodulation of the DUT signal. Also, thermal effects caused by the environment or the optical power itself can change the refractive index in waveguides, altering light propagation paths and leading to performance instability. Furthermore, due to processing uniformity issues, parameters such as waveguide dimensions, the refractive index, and the above properties can vary from chip to chip. These variations affect the performance consistency of devices, making them difficult to achieve uniformity in large-scale production. However, with the help of digital demodulation, all these effects can be calibrated and compensated in this work.

To mitigate signal interference caused by ghost reflectance or scattering signals, a secondary quadrature demodulation network is implemented with the primary network to capture similar characteristics. By following digital decoding and appropriate filtering, correlation between signals from the first and second quadrature networks is computed. This correlation is then used to compensate the measurement signal, resulting in a more accurate outcome. To further compensate the temperature effect, a third quadrature demodulation network set with a distinct reference arm delay line length is included in the chip. As temperature or environmental variations impact the refractive index and other properties of the delay line, discrepancies in the measurement results between the second and third demodulators can reflect these changes. Through signal processing and analysis, shifts in the refractive index can be compensated in real time to mitigate the nonlinear interference caused by the temperature and other factors.

With these aforementioned efforts, a chip-based laser vibrometer with a homodyne detection configuration is able to achieve the same performance as a high-performance conventional laser vibrometer with a heterodyne detection configuration. It is worth mentioning that a heterodyne detection configuration can also be adopted in photonic integration. The current design strikes a balance between power consumption, cost, and performance, and there is a substantial potential for further performance enhancements.

## 3. Performance of Laser Vibrometers by Integrated Photonics

### 3.1. Advantages of Integrated Photonics-Based Laser Vibrometers

With the rapid evolution of the telecommunication industry, silicon photonics and integrated optical technologies have matured significantly due to their high performance, scalability, and cost effectiveness. In the future, it will also be possible to integrate optical and electronic components into the same chip, enabling seamless data transmission, signal processing, and other functions in a unified optoelectronic circuit. Compared to discrete optics, integrated optics can more easily and effectively achieve the features depicted in Figure 3. For instance, the 90° mixing network by integrated photonics has the following advantages: (1) Compactness: integrated optics facilitates the integration of multiple optical components, such as beamsplitters, phase shifters, and PDs, into a compact chip, greatly minimizing the system size. The component is only a few hundred micrometers long and wide in size. Thus, the 90° mixing network composed mainly of optical waveguide elements can be densely packed in an integrated optical chip, yielding a lighter and smaller overall system. (2) Precise alignment: aligning individual components in discrete optical systems is an error-prone and time-intensive process. Integrated optical chips leverage nanoscale manufacturing to directly etch precise optical waveguides, couplers, and other components onto the chip surface, ensuring optical path stability and minimizing mechanical alignment requirements. (3) Environmental stability: discrete optical systems are vulnerable to environmental variables such as temperature, vibration, and humidity. In contrast, integrated optical systems secure all components on a single substrate, resulting in a robust and stable structure that resists external environmental changes and boosts system reliability. Although the waveguide-based design is still susceptible to temperature change, as discussed in Section 2.3, the effect due to temperature-induced index variation can be mitigated in the design. Also, a thermoelectric cooler can be easily applied in the design due to the compactness of the integrated circuit. (4) Manufacturing consistency: integrated optical devices employ semiconductor manufacturing processes that enable mass production and guarantee uniformity across demodulators. However, discrete optical systems require manual assembly and adjustment, often resulting in inconsistency among units.

**Figure 4:**
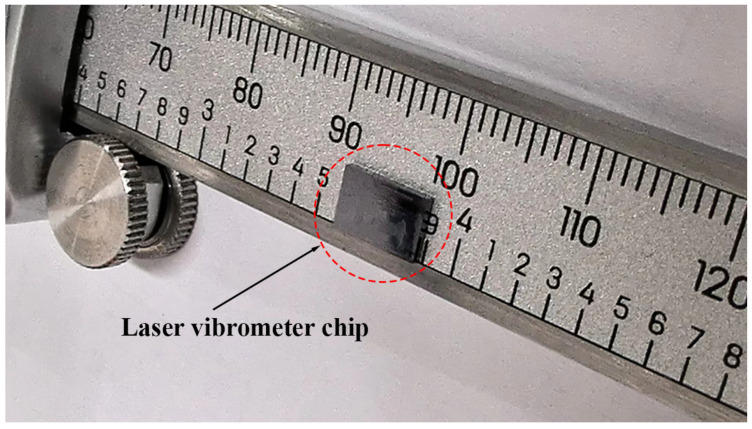
Actual size of the laser vibrometer chip proposed in this work, which is 7 mm × 5 mm.

The actual size of the laser vibrometer chip proposed in this work is 7 mm × 5 mm, as shown in Figure 4. With the progress of advanced photonic integration technologies, the chip’s size can be further reduced by 10–100 times. Integrated optics using nanoscale manufacturing can incorporate additional components and features, such as modulators and filters, directly in the same chip. Individual functional modules can be combined with other components to deliver advanced functionality while reducing signal transmission loss. For instance, the laser vibrometer chip detailed in this work also includes a frequency-modulated continuous wave module, allowing for the high-precision positioning of measurement points during scanning. Furthermore, mass production and manufacturing consistency enable integrated optics to significantly reduce the production costs per chip and lower the labor and maintenance expenses. Within the same chip, measurement units can be easily replicated to achieve multi-channel measurements while ensuring consistent results across all channels. Lastly, integrated photonics can be massively produced on wafers as other integrated circuits are made. This cuts down the material cost of a vibrometer dramatically. Also, it is much simpler to assemble a vibrometer using integrated photonics than a vibrometer using discrete optics. Thus, the overall manufacture cost of a vibrometer is considerably reduced.

### 3.2. Performance of Laser Vibrometers Based on Integrated Optics

In this work, a typical telecom commodity laser is used with a laser linewidth around 1 MHz and a laser chip output power as 20 mW under a typical driving current. The laser is hermetically packaged together with PICs. Although PICs can be fabricated on any platforms, a material platform with a lower thermal coefficient is more helpful to mitigate the thermal effect. For example, the silicon nitride (SiN) material has a thermal coefficient that is ten times lower than that of the SOI material. Therefore, the work introduced here is fabricated on a variant of the SiN material, although it has a higher core index and a larger design bending radius than the SOI material. Compared to other platforms, the SiN platform is a more balanced photonic integration platform for optical sensing applications: (1) its reasonable high-index contrast ratio enables it to have a miniatured chip size; (2) it has low insertion loss with 0.04 dB/cm waveguide propagation loss; (3) it has fewer waveguide defects so that there are fewer ghost reflectance signals to the receiver compared to the SOI platform; and (4) it has mature fabrication to assure photonic uniformity across the wafer. Thus, the platform allows one to have a relatively complicated design into a chip, such as a second quadrature network together with a delay line for common mode reduction. There can be index variations across wafers and even among different locations on a single wafer, which results in phase errors on the 90° hybrid or any phase-sensitive components in the design. Two techniques are used in the design of laser vibrometers proposed in this work to achieve the final performance. The first is to mitigate processing errors before digital decoding through the photonic design [24]. The second is to compensate the *I*/*Q* imbalance and the phase shift induced during manufacturing in digital decoding [23].

**Figure 5:**
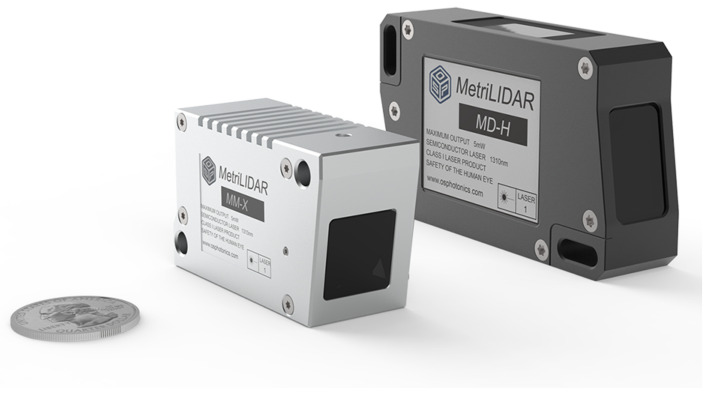
Two laser vibrometers developed using integrated optical chips.

Two laser vibrometers developed using the integrated optical chip are shown in Figure 5; the left one is the smallest laser vibrometer ever recorded that measures only 58 mm × 34 mm × 22 mm in size and weighs 86 g. It features a full metal casing and an IP67 environmental protection rating, ensuring reliable operations even in harsh environments. Its peak power consumption is less than 1.5 watt at the 5 volt DC input; thus, this device can be powered by a lithium battery with a typical 2000 mAh for 3–4 h usage. Its lightweight and low-power consumption facilitate it to be integrated into UAVs and robotic platforms, making non-contact laser vibration measurement a practical component for drone and robotic sensing.

**Figure 6:**
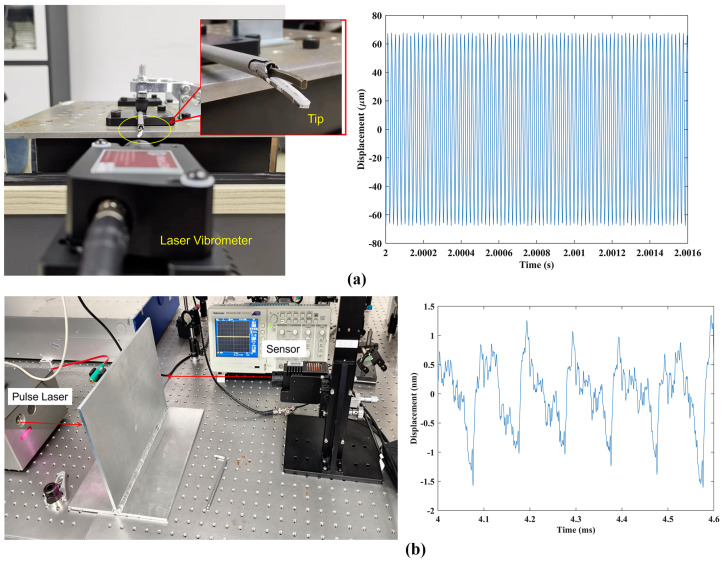
(**a**) Vibration of a medical scalpel tip; and (**b**) the measurement of ultrasonic waves induced by a high-energy laser pulse.

The right device in Figure 5, which is referred to as MotionGo (OmniSensing Photonics LLC, Columbia, MD, USA) is used in the next section for performance validation. The vibrometer can work at a sampling rate of 5 million samples per second (Msps). Because it is based on the homodyne laser interferometer theory, the device can measure vibration signals ranging from DC to 2.5 MHz. It is equipped with a versatile lens system, allowing one to switch focusing lenses as needed for a detection range from 4 cm to 100 m. Each lens includes manual focus adjustment to optimize the receipt of optical signals under different measurement distances. As an intelligent measurement unit, it supports large-scale networking with both synchronous input and output interfaces, facilitating synchronized network measurement. It delivers outstanding noise performance, capable of measuring distances up to 100 m and detecting vibrations up to 20 m/s. The performance specifications of MotionGo are summarized in Table 1. In Figure 6, two sets of data were measured using the vibrometer. Figure 6a represents the vibration of a medical scalpel tip. This scalpel operates at a frequency of 49.2 kHz, with a maximum tip displacement of 126.6 mm. Consequently, the vibration velocity is calculated to be 19.57 m/s. These results demonstrate the vibrometer’s capability to measure vibrations across an extensive velocity range. It is important to note that the proposed vibrometer operates at 5 Msps. If it were operated at a typical 100 Msps, like other conventional vibrometers, its measurement range could be extended up to 20 times larger. Figure 6b illustrates the measurement of ultrasonic waves induced by a high-energy laser pulse. Despite the vibrations being as weak as at the sub-nanometer level, the vibrometer successfully detects the pulse trains of the vibrations in real-time, maintaining a noise floor of less than 0.1 nm.

## 4. Experimental Validation

### 4.1. Measurement Repeatability

**Figure 7:**
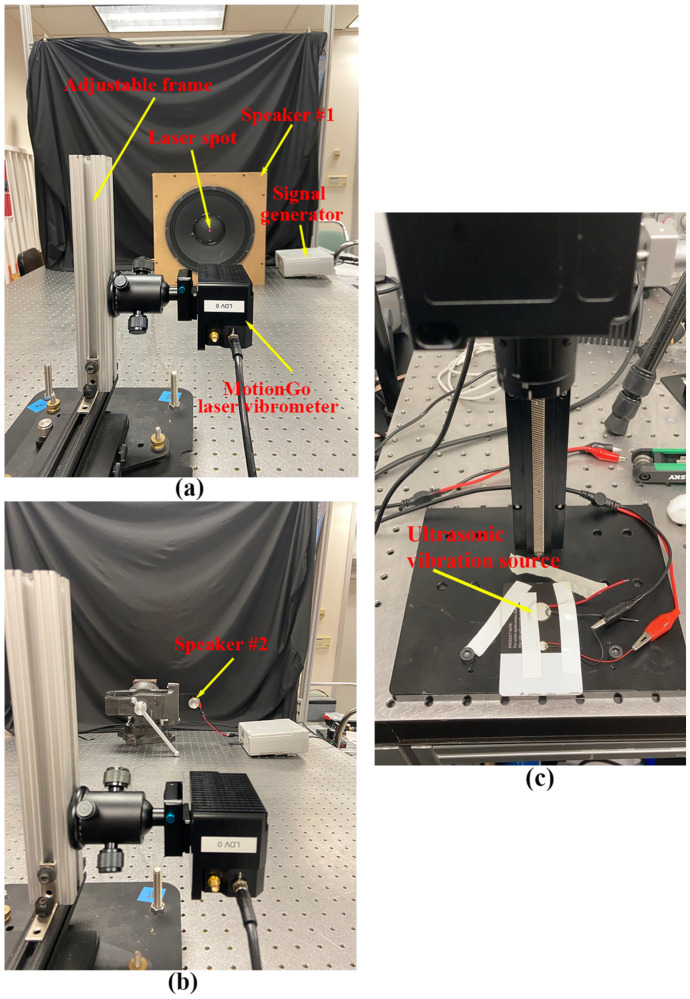
Experimental setups of the measurement repeatability validation of the proposed laser vibrometer: (**a**) a speaker under excitation frequencies of 0.1 Hz–1000 Hz; (**b**) another speaker under excitation frequencies of 5000 Hz and 20,000 Hz; and (**c**) an ultrasonic vibration source under an excitation frequency of 1,000,000 Hz.

**Figure 8:**
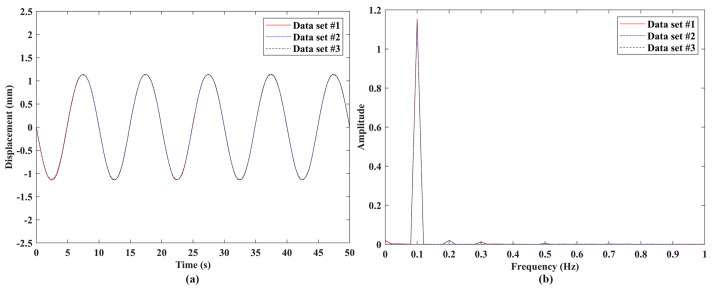
Vibrations of the speaker #1 under sinusoidal excitation with a frequency of 0.1 Hz: responses from three independent datasets with the same length in the (**a**) time domain and (**b**) frequency domain.

**Figure 9:**
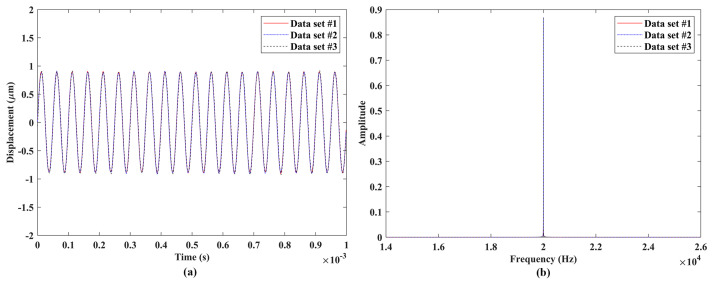
Vibrations of the speaker #2 under sinusoidal excitation with a frequency of 20,000 Hz: responses from three independent datasets with the same length in the (**a**) time domain and (**b**) frequency domain.

**Figure 10:**
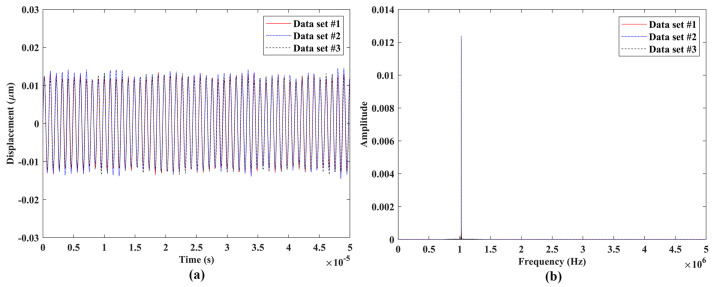
Vibrations of the ultrasonic vibration source under sinusoidal excitation with a frequency of 1,000,000 Hz: responses from three independent datasets with the same length in the (**a**) time domain and (**b**) frequency domain.

Experimental validation of the measurement repeatability of the proposed laser vibrometer was conducted through comparisons among vibration responses from three independent measurements of a DUT. The experimental setups of measurement repeatability validation are shown in Figure 7, where the three DUTs were used for measurements with vibration frequencies much lower than, close to, and within the ultrasonic range, respectively. The laser vibrometer was attached to an adjustable frame in measurements to ensure that its laser beam was perpendicular to the surfaces of the DUTs and its laser spot was located at the centers of the DUTs. A signal generator was used to provide sinusoidal excitation to the DUTs. A speaker #1 shown in Figure 7a was used as the DUT that was under sinusoidal excitations with frequencies of 0.1 Hz, 1 Hz, 10 Hz, 100 Hz, and 1000 Hz, which were much lower than the ultrasonic range. A speaker #2 that was smaller than the speaker #1, as shown in Figure 7b, was used as the DUT that was under sinusoidal excitations with frequencies of 5000 Hz and 20,000 Hz, which were close to the ultrasonic range. An ultrasonic vibration source shown in Figure 7c was used as the DUT that was under sinusoidal excitation with a frequency of 1,000,000 Hz, which was within the ultrasonic range. For each excitation frequency, three independent tests were performed with the same data length. The sample results of the three DUTs are shown in Figure 8, Figure 9 and Figure 10, respectively. One could see that the measured vibration responses from the three datasets for each excitation frequency had good agreement with each other in both the time and frequency domains. To further check the measurement repeatability, the peak values of the vibrations of the DUTs under various excitation frequencies were extracted from their frequency spectra and listed in Table 2, where the percentage differences among them were calculated by using each dataset #1 as a reference. One could see that the maximum difference was lower than 0.5%, meaning that the proposed laser vibrometer could measure vibrations in a wide frequency range with a high level of repeatability.

### 4.2. Measurement Accuracy

**Figure 11:**
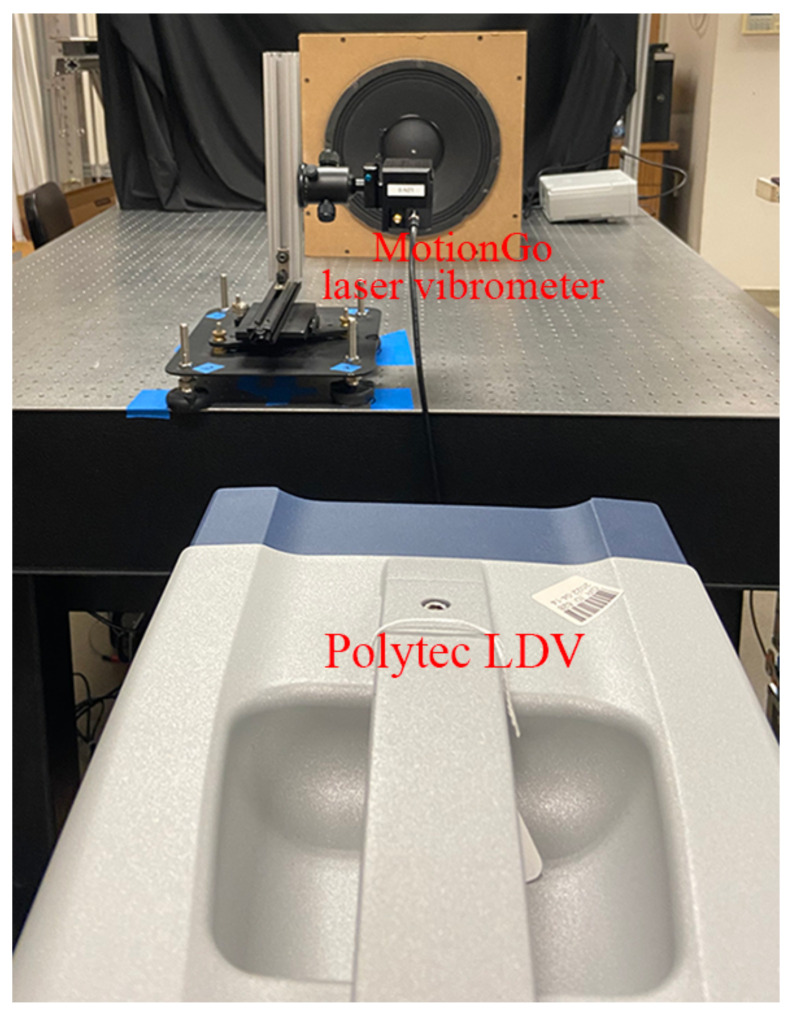
Experimental setup of the measurement accuracy validation of the proposed laser vibrometer, where a Polytec LDV was used as a reference.

**Figure 12:**
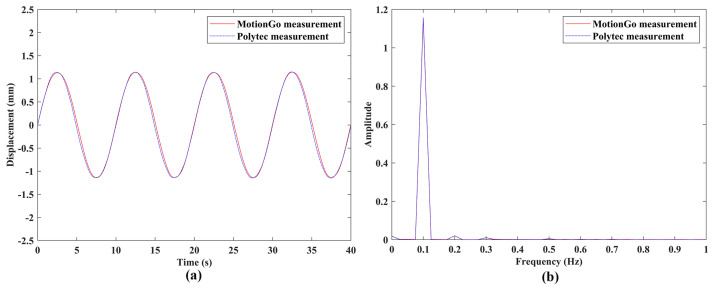
Vibrations of the speaker #1 under sinusoidal excitation with a frequency of 0.1 Hz: responses from the two independent measurement systems with the same length in the (**a**) time domain and (**b**) frequency domain.

**Figure 13:**
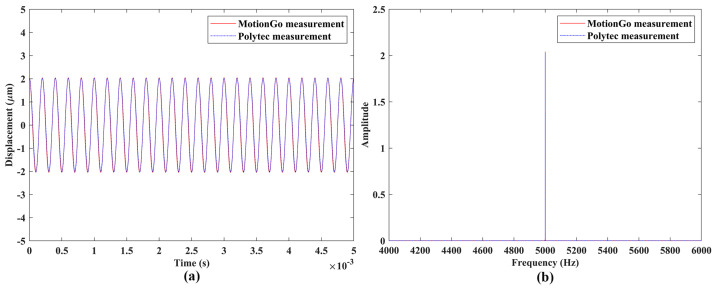
Vibrations of the speaker #2 under sinusoidal excitation with a frequency of 5000 Hz: responses from the two independent measurement systems with the same length in the (**a**) time domain and (**b**) frequency domain.

Experimental validation of the measurement accuracy of the proposed laser vibrometer was conducted through comparisons between vibration responses from the measurements of a DUT using two independent measurement systems; vibration response measured by a Polytec LDV (PSV-500) (Polytec GmbH, Waldbronn, Baden-Württemberg, Germany), as shown in Figure 11, was used as a reference. Speakers #1 and #2 were used in this work to provide vibrations with frequencies up to 5000 Hz, since the Polytec LDV used in this work had a limited maximum sampling rate and was not an LDV designed for measuring ultrasonic vibration. For each excitation frequency, the tests performed by the two measurement systems had the same data length. The sample results of the two DUTs were shown in Figure 12 and Figure 13. Note that the responses measured by the proposed laser vibrometer were in terms of displacement, while those measured by the Polytec LDV were in terms of velocity. Therefore, velocity responses from the Polytec LDV were integrated to be displacement ones prior to comparison. One can see that the measured vibration responses from the two systems for each excitation frequency had good agreement with each other in both the time and frequency domains. To further check the measurement accuracy, the peak values of the vibrations of the DUTs under various excitation frequencies were extracted from their frequency spectra and listed in Table 3. One could see that the maximum difference was lower than 1%, meaning that the proposed laser vibrometer could measure vibrations in0 a wide frequency range with a high level of accuracy.

### 4.3. Robustness to Test Surface Conditions

**Figure 14:**
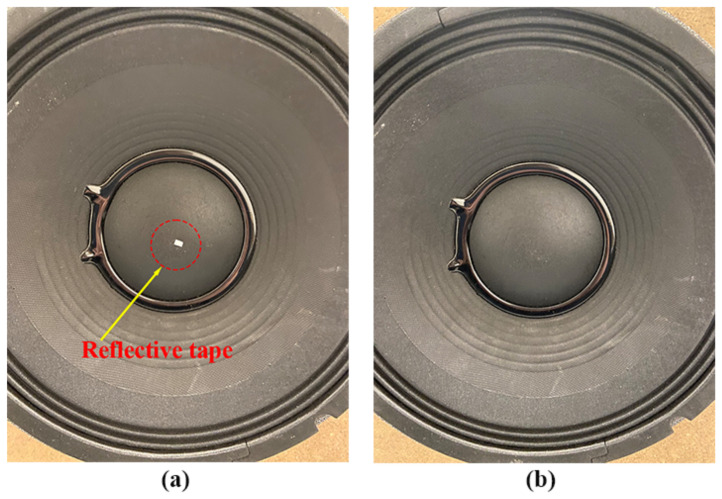
Experimental setup for assessing the robustness of the proposed laser vibrometer to test surface conditions using the speaker #1 with (**a**) a surface enhanced by a reflective tape, and (**b**) a natural surface.

This section aimed to assess the performance of the proposed laser vibrometer for different test surface conditions, where the SNR was used as an indicator. The experimental setup is shown in Figure 14, where the left subfigure was the speaker #1 with a piece of reflective tape on its surface and the right subfigure was the speaker #1 with its natural surface. It could be seen that the natural surface of the speaker #1 was dark and rough, meaning that it had weak reflection. For a conventional LDV without a signal enhancement technique, attaching a reflective tape on the test structure surface was an important and necessary step in the test preparation stage. A signal enhancement technique, such as the QTec technology developed by Polytec, usually used multiple channels, while the laser vibrometer proposed in this work was based on a single-channel detection. For fair comparison purposes, a conventional Polytec LDV without the QTec technology signal enhancement was used in this comparison.

**Figure 15:**
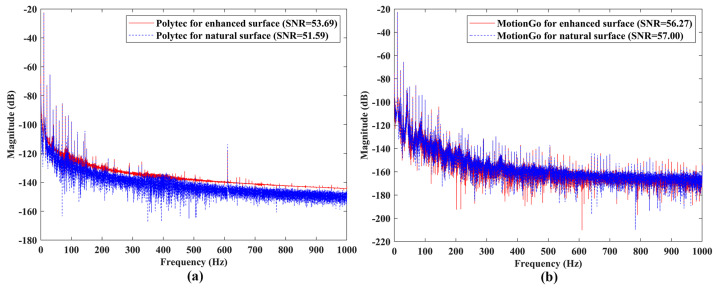
SNRs of measured responses of the speaker #1 with enhanced and natural surfaces under sinusoidal excitation with a frequency of 10 Hz using (**a**) the Polytec LDV, and (**b**) the proposed laser vibrometer.

**Figure 16:**
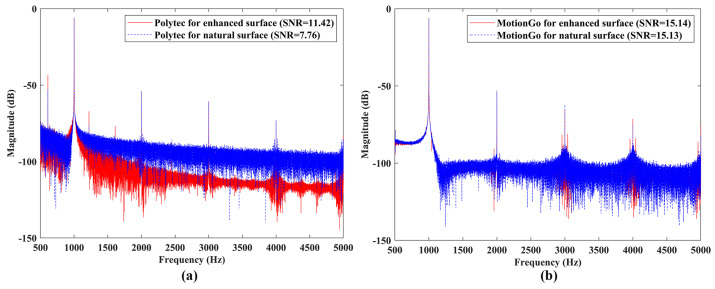
SNRs of measured responses of the speaker #1 with enhanced and natural surfaces under sinusoidal excitation with a frequency of 1000 Hz by using (**a**) the Polytec LDV; and (**b**) the proposed laser vibrometer.

Vibration responses of the speaker #1 under sinusoidal excitations with frequencies of 10 Hz and 1000 Hz, measured by the Polytec LDV, which was a conventional model without the QTec technology, were shown in Figure 15a and Figure 16a, respectively. One could see that the SNRs of the responses slightly decreased from 53.69 to 51.59 for the case of 10 Hz excitation, and significantly decreased from 11.42 to 7.46 for the case of 1000 Hz excitation, when removing the reflective tape. Vibration responses of the speaker #1 under sinusoidal excitations with frequencies of 10 Hz and 1000 Hz, measured by the proposed laser vibrometer, are shown in Figure 15b and Figure 16b, respectively. The SNRs of the responses remained approximately constant for enhanced and natural surfaces, meaning that the proposed laser vibrometer had a good robustness to the test surface conditions. Note that the SNR results from the two measurement systems were not compared to each other, as they had different measurement conditions, such as measurement distances and optical diameters.

## 5. Conclusions

This work proposes a novel chip-based high-precision laser vibrometer that can directly measure the vibration displacement of a structure through the phase demodulation method. The feasibility for the productization and the features for the vibration measurement of the proposed miniatured laser vibrometer are discussed in this work. Some conclusions are listed as follows:

1. The laser vibrometer proposed in this work incorporates two or more sets of quadrature demodulation networks in the design. One quadrature demodulation network serves as a main *I*/*Q* demodulator for signal measurement. A reference quadrature demodulation network can capture the phase and amplitude instability of the laser. Correlation between the signals from the first and second quadrature networks can be computed to mitigate signal interference caused by ghost reflectance or scattering signals. A third quadrature demodulation network set with a distinct reference arm delay line length can be included to further compensate the temperature effect by capturing temperature or environmental variations that impact its delay line.

2. Experimental validation of the measurement repeatability of the proposed laser vibrometer was conducted through comparisons among the vibration responses from the three independent measurements of the DUTs under sinusoidal excitations with frequencies from 0.1 Hz to 1,000,000 Hz. The maximum difference among measurements is lower than 0.5%, meaning that the proposed laser vibrometer can measure vibrations in a wide frequency range with a high level of repeatability.

3. Experimental validation of the measurement accuracy of the proposed laser vibrometer was conducted through comparisons between vibration responses from measurements of the DUTs using the proposed laser vibrometer and a Polytec LDV. The maximum difference between the results from the two measurement systems was lower than 1%, meaning that the proposed laser vibrometer could measure vibrations in a wide frequency range with a high level of accuracy.

4. SNRs of responses measured by the proposed laser vibrometer could remain approximately constant for surfaces with and without reflective tapes, meaning that it had better robustness to test surface conditions than a conventional LDV.

## Figures and Tables

**Table 1:** Performance specifications of MotionGo.

Parameter (Unit):	Value	Parameter (Unit):	Value
Measurement distance (m)	0.025–100	Measurement frequency range (MHz)	DC-2.5
Noise density $\left(\mathrm{pm}/\sqrt{\mathrm{Hz}}\right)$	<0.1	Speed range (m/s)	>20
Displacement resolution (nm)	0.01	Displacement repeatability (nm) (100 kHz receiver bandwidth)	<0.1
Laser specification	1310 nm measurement, 655 mm indicator	Measurement laser output power (mW)	<5
Laser safety class	Class I	Indicator laser output power	Class I or adjustable

**Table 2:** Peak values of vibrations of the DUTs under various excitation frequencies, where three independent datasets were measured for each excitation frequency and differences among them were calculated by using each dataset #1 as a reference (unit: μm).

DUT	Excitation Frequency (Hz)	Dataset #1	Dataset #2	Dataset #3	Differences
Speaker #1	0.1	1154.4	1149.7	1149.3	0.4% & 0.4%
	1	116.1	116.2	116.3	0.1% & 0.2%
	10	75.3	75.2	75.4	0.1% & 0.1%
	100	30.3	30.3	30.3	0 & 0
	1000	$4.9\times 10^{-1}$	$4.9\times 10^{-1}$	$4.9\times 10^{-1}$	0 & 0
Speaker #2	5000	$1.8\times 10^{-1}$	$1.8\times 10^{-1}$	$1.8\times 10^{-1}$	0 & 0
	20,000	$8.7\times 10^{-1}$	$8.7\times 10^{-1}$	$8.7\times 10^{-1}$	0 & 0
Ultrasonic vibration source	1,000,000	$1.2\times 10^{-2}$	$1.2\times 10^{-2}$	$1.2\times 10^{-2}$	0 & 0

**Table 3:** Peak values of vibrations of the DUTs under various excitation frequencies, where vibration responses were measured for each excitation frequency using the two independent measurement systems and differences among them were calculated by using each data from the Polytec LDV as a reference (unit: μm).

DUT	Excitation Frequency (Hz)	Polytec Measurement	MotionGo Measurement	Differences
Speaker #1	0.1	1157.99	1157.97	<<0.01%
	1	600.95	595.17	0.96%
	10	75.50	74.97	0.70%
	100	44.67	44.61	0.13%
	1000	$4.97\times 10^{-1}$	$4.95\times 10^{-1}$	0.40%
Speaker #2	5000	2.03	2.04	0.49%

## Data Availability

Dataset available upon request from the authors.
